# Nanoform of Phospholipid Composition: Investigation of the Morphological Features by Atomic Force Microscopy

**DOI:** 10.3390/ijms242015338

**Published:** 2023-10-19

**Authors:** Sergey V. Kraevsky, Irina A. Ivanova, Sergey L. Kanashenko, Ivan D. Shumov, Ilya A. Ryazantsev, Yulia A. Tereshkina, Lyubov V. Kostryukova, Yulia A. Romashova, Tatyana O. Pleshakova

**Affiliations:** Institute of Biomedical Chemistry, Pogodinskaya Str., 10, Moscow 119121, Russia; i.a.ivanova@bk.ru (I.A.I.); serkanash@mail.ru (S.L.K.); shum230988@mail.ru (I.D.S.); ilryazancev@yandex.ru (I.A.R.); burova13@gmail.com (Y.A.T.); kostryukova87@gmail.com (L.V.K.); juliademich@gmail.com (Y.A.R.); topleshakova@yandex.ru (T.O.P.)

**Keywords:** atomic force microscopy, dynamic light scattering, nanophospholipid composition, phosphatidylcholine, vesicle, phospholipid membrane

## Abstract

Morphological features of the nanoform of a phospholipid composition (NFPh), which can be used as an individual pharmaceutic agent or as a platform for designing drug delivery systems, have been studied using atomic force microscopy (AFM). NFPh has been developed, and its characteristics have been investigated using conventional drug analysis methods, including the determination of the mean diameter of nanosized vesicles in the emulsion via dynamic light scattering (DLS). Using DLS, the mean diameter of the vesicles was found to be ~20 nm. AFM imaging of the surface has revealed four types of objects related to NFPh: (1) compact objects; (2) layer fragments; (3) lamellar structures; and (4) combined objects containing the compact and extended parts. For type (4) objects, it has been found that the geometric ratio of the volume of the convex part to the total area of the entire object is constant. It has been proposed that these objects formed owing to fusion of vesicles of the same size (with the same surface-to-volume ratio). It has been shown that this is possible for vesicles with diameters of 20 nm. This diameter is in good coincidence with the value obtained using DLS.

## 1. Introduction

Over the past decades, the intense development of nanotechnologies aiming to comprehensively study properties of nanosized objects of various nature, as well as the potential of their medical applications, has given rise to new disciplines: nanomedicine and nanobiology [1,2,3,4,5,6,7,8]. Owing to their properties, nanosized objects, such as nanoparticles, are used in both pharmacology and medicine for diagnostic [9] and therapeutic applications [10,11]. By introducing the nanomaterials into biomedicine, specialized diagnostic tools (contrast reagents and analytical instruments) and nanosized drug delivery systems have been developed [12].

The use of phospholipid vesicles as drug carriers has many advantages—similar in structure to the cell membrane, high biocompatibility, and low toxicity [13]. At that, soy phospholipids are the most frequently used non-allergic and biodegradable components of vesicles in nanomedicine [14,15]. These phospholipids mainly include phosphatidyl choline—the major component of cell membranes and blood plasma lipoproteins. 

Chemically, phosphatidylcholine represents a zwitterion, whose polar end bears some “excess” negative charge, owing to the presence of a phosphate group, and some “excess” positive charge borne by the nitrogen atom of the choline group. Thus, in phospholipid vesicles, a drug can be incorporated into phospholipid nanoparticles (depending on the pH of the medium, particle type, and properties of the drug to be incorporated) in the inner region (nucleus), in the phospholipid bilayer, and on the surface of the vesicles [16]. Many drug substances and biologically active compounds are known to be poorly soluble or completely insoluble in water (hydrophobic). This leads to their low bioavailability. In order to improve the effectiveness of these compounds, their solubility needs to be improved. The method of high-pressure homogenization is one of approaches for improving their solubility [17]. Phospholipid nanoparticles enhance the bioavailability of the incorporated agent and reduce its clearance. Furthermore, the surface of phospholipid-based nanoparticles can be easily modified with various functional groups, including vector molecules [18,19]. This is why phospholipid nanoparticles are considered as the basis of nanopharmaceuticals in many investigations [20,21,22].

The size of nanopharmaceuticals is one of the crucial factors which determines their behavior in the body. Controlling the size of drug-carrying nanoparticles is important, since the in vivo pharmacokinetics of nanoparticles directly depends on their size [23]. The latter affects their distribution in the living organism and the rate of their internalization by cells of the mononuclear phagocyte system [24]. A relatively small reduction in particle size prolongs their circulation and enhances their ability to reach specific target tissues [25].

In order to determine the size of phospholipid vesicles, their homogeneity, and their morphology, size exclusion chromatography (SEC) [26], field-flow fractionation [27], static or dynamic light scattering (DLS) [28,29], transmission electron microscopy (TEM) [30] and atomic force microscopy (AFM) [31] are employed. DLS is commonly employed for the characterization of pharmaceuticals. And in the present study, AFM has been employed in parallel with DLS in order to characterize a nanoform of phospholipid composition (NFPh). 

In the macroscopic DLS method, the mean hydrodynamic diameter of nanoparticles in an aqueous colloidal system (emulsion or suspension) is measured based on the intensity of light scattered by these particles, which are considered to be spherical. The key advantages of DLS include its simplicity and applicability to nanoparticles suspended in a buffered aqueous medium. The mean diameters in the particle size distribution, obtained using DLS, can, however, be overestimated; the intensity of light scattered by a spherical particle is directly proportional to the sixth power of its diameter, so the intensity of light scattered by smaller particles is lost due to the influence of the background signal [32].

Hoo et al. [32] compared the results obtained for polystyrene nanoparticles using DLS and using AFM. These authors studied the size distributions of standard polystyrene nanoparticles of two different sizes: 20 nm and 100 nm. The reference (nominal) value was the one determined using transmission electron microscopy (TEM). For colloidal systems containing one type of nanoparticles (i.e., the nanoparticles of one and the same size), their average size measured using DLS and using AFM was shown to coincide with the reference TEM value within the experimental error. However, for colloidal systems containing two types of particles, bimodal distributions were successfully characterized by AFM, but the results obtained using DLS were shifted towards larger values. Hoo et al. claimed that characterization of nanoparticles using AFM using Nano Rule+^TM^ software (Pacific Nanotechnology, Inc., Santa Clara, CA, USA [32]) for automated analysis is accurate and rapid, thus being preferable over DLS for non-monodisperse colloidal systems. The results obtained by these authors [32] indicate that analytical methods used for studying nanopharmaceuticals should be selected very carefully.

The advantages of using AFM in the development of nanopharmaceuticals were thoroughly discussed by Sitterberg et al. in their review [33]. These authors unveiled the features of using this method during the development, characterization, and delivery of nanoscale drug delivery systems such as nanoparticles, liposomes, and polymeric particles. They also stated that AFM allows one to avoid a laborious and potentially contaminating sample preparation procedure for studying nanosized objects. Furthermore, AFM studies can be performed in a controlled environment without staining or drying. Moreover, the non-contact mode of AFM allows one to examine soft samples, thus providing minimum impact on the sample (which may cause its subsequent alteration). The phase imaging AFM mode provides access to information beyond sample topography.

It should, however, be noted that in AFM, the nanosized objects under study—including vesicles—should be adsorbed on the surface of a solid substrate. At that, in AFM, images of surface-adsorbed vesicles—not their structure in aqueous solution—are obtained [31]. Adsorption onto the surface can be accompanied by vesicles’ fusion and rupture [34]. This fact is widely used in AFM studies of membranes [35,36]. Thus, one of the aims of our present study was the visualization of compact objects among incompletely formed layers and the patterns of such a fusion. 

In the present study, we have conducted NFPh preparation experiments, and we have determined its characteristics using both the macroscopic methods and AFM. We have selected soybean phosphatidylcholine (Lipoid S100) as a starting component for NFPh development. Such a highly dispersed nanosystem represents a universal model for delivery of numerous different biologically active compounds, including hydrophobic ones [17].

## 2. Results

### 2.1. NFPh Characterization by Macroscopic Methods

**Figure 1:**
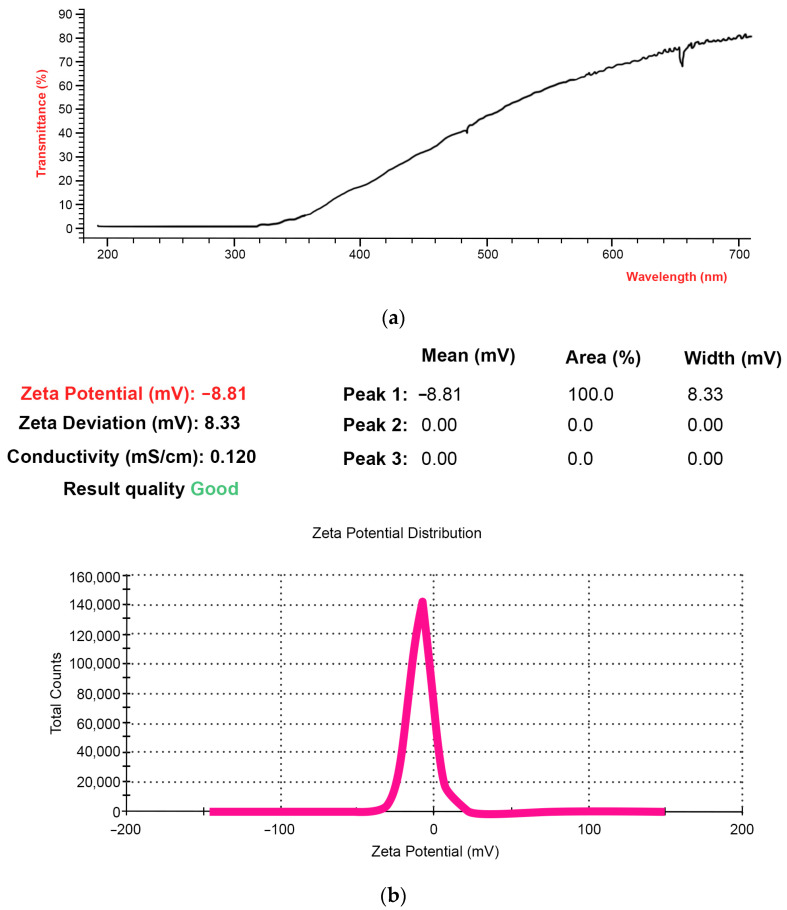
Light transmittance (**a**) and zeta potential (**b**) of the prepared phospholipid emulsion (NFPh). In the legend of panel (**b**), the zeta potential is highlighted in red.

Based on the previously elaborated technology for developing a phospholipid delivery system for drug incorporation, we obtained the nanoform of phospholipid composition NFPh. The latter comprised two components: soybean phosphatidylcholine and disodium glycyrrhizinate (which was used as a detergent providing stability of the nanoparticles). As a result, a homogeneous ultrafine emulsion has been prepared. Once the emulsion sample had been prepared, the pH, transparency (light transmittance), and zeta potential of the emulsion particles were studied. The pH of the emulsion was 7.33 (neutral). Figure 1 displays the emulsion transmittance spectrum (Figure 1a) and the resulting zeta potential value (Figure 1b).

The light transmittance of the emulsion was 77.08 ± 0.05% (Figure 1a). This indirectly indicates that the emulsion contained extremely small particles. The presence of large particles in the emulsion can cause the abrupt spontaneous aggregation of phospholipid particles, leading to opacification of the emulsion and reducing its transmittance.

The zeta potential is another important property of the resulting phospholipid nanoform. The zeta potential value characterizes the aggregate stability of the emulsion particles: the higher its absolute value is, the larger the surface charge of the particles is. The latter provides electrostatic stabilization of the particles, increasing electrostatic repulsion between them, thus preventing their aggregation. As described in [37], zeta-potential values greater than +30 mV and lower than −30 mV indicate conditions of stability, while the values between −30 mV and +30 mV indicate conditions of stability favoring the aggregation, coagulation, and flocculation of the particles. The zeta potential of the phospholipid nanoform was −8.8 ± 0.8 mV (Figure 1b), indicating that the resulting emulsion was insufficiently stable during long-term storage. In order to increase the emulsion stability, stabilizers, such as maltose, can be employed [17]. However, the presence of maltose in the samples should be avoided if the samples are intended to be studied using AFM. This is the reason why in our reported experiments, a freshly prepared stabilizer-free emulsion has been used.

### 2.2. Characteristics of NFPh Measured by DLS

**Figure 2:**
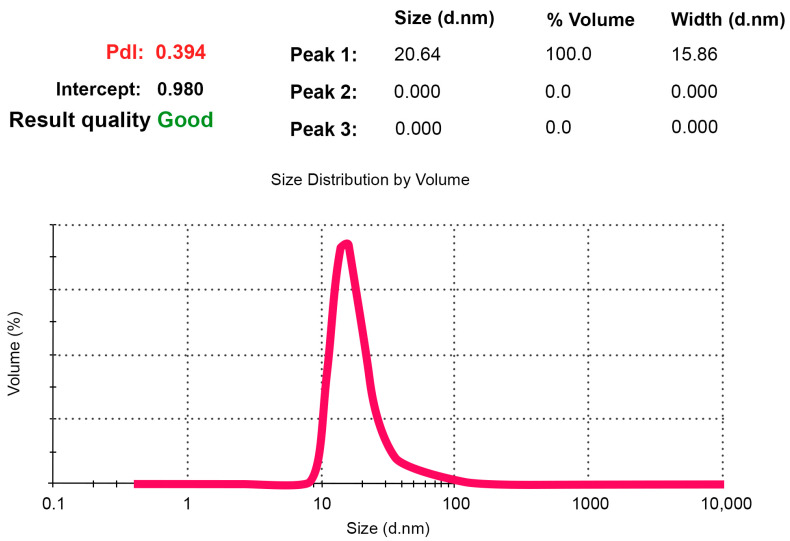
Particle size distribution in the NFPh emulsion obtained using DLS. The polydispersity index is highlighted in red.

Figure 2 displays the size distribution of NFPh particles measured using DLS.

As was noted in the Introduction, the particle size is one of the key parameters of nanopharmaceuticals, which affects the distribution of the particles in the body and the rate of internalization of larger particles by mononuclear phagocyte system cells [38]. According to the DLS data obtained in our experiments, over 95% of the NFPh emulsion volume is represented by particles of a diameter up to 50 nm. The size distribution peak corresponds to ~21 nm. Such a small (≤50 nm) diameter of nanoparticles reduces their internalization by the mononuclear phagocyte system (MPS)—the natural mechanism ensuring protection against pathogens entering the body. This allows one to use NFPh as a delivery system for a number of drug substances of various therapeutic categories.

In phospholipid emulsions, nanoparticles within one system can be heterogeneous in terms of their size (i.e., polydisperse). A parameter called the polydispersity index (PDI) is used to estimate the degree of particle fineness in the composition. It characterizes the heterogeneity of the particle size distribution and is calculated using a two-parameter cumulant analysis (using a logarithmic function). It is a dimensionless index, which was initially used in polymer chemistry. In theory, it varies from 0.0 for a completely homogeneous particle mixture to 1.0 for highly dispersed mixtures [14]. In drug delivery systems based on lipid vesicles, a PDI ≤ 0.3 is considered to be acceptable, indicating that the vesicle population is relatively homogeneous [39]. The PDI value was 0.39, indirectly indicating that the emulsion contained a certain amount of potentially aggregated molecules (<5%). This is consistent with the estimation of the critical micelle concentration of phosphatidylcholine (see Figure S1 in Supplementary Materials File).

### 2.3. Characteristics of NFPh Measured by AFM

**Figure 3:**
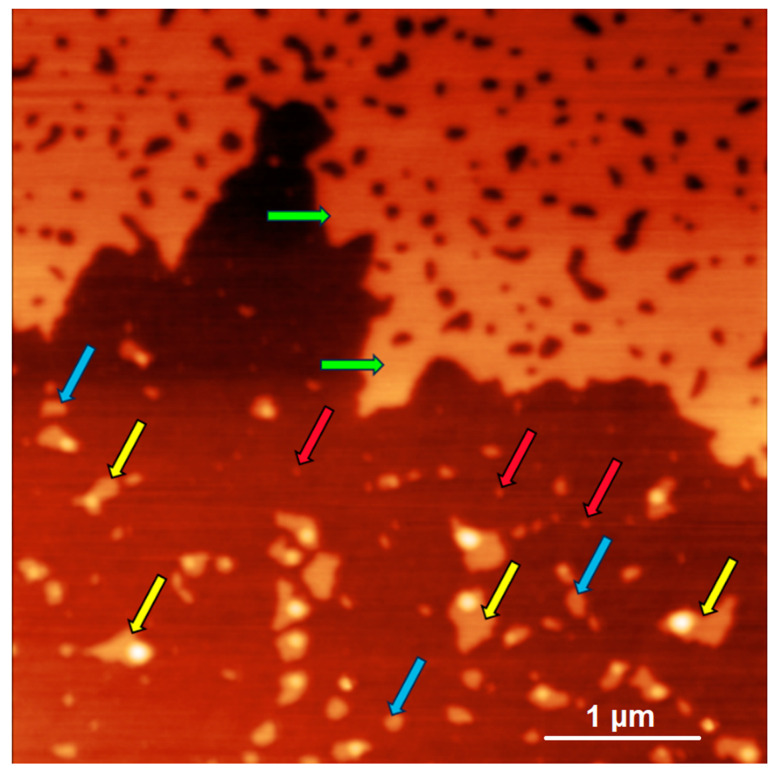
Typical AFM image of the mica substrate surface after incubation in NFPh emulsion. The surface was visualized immediately after sample preparation. Arrows indicate the examples of different types of visualized objects: type#1 (red arrow)—compact objects of 2–3 nm height, with a diameter less than 100 nm; type#2 (blue arrow)—extended flat objects of ~5 nm thickness, with a surface area of >10^4^ nm^2^; type#3 (green arrow)—extended objects of 5–7 nm thickness; and type#4 (yellow arrow)—combined objects containing a compact part of 7–30 nm height, which protrudes above an extended flat part of 2–5 nm thickness. Frame size, 5 × 5 µm^2^; Z scale: 0–16 nm.

Figure 3 displays a typical AFM image of the mica surface after its incubation in the NFPh emulsion.

In Figure 3, one can observe several types of objects on the substrate surface. Compact objects of 2 to 3 nm height were attributed to type#1 objects. Their diameter was ≤100 nm. In Figure 3, examples of this type of object are pointed to by red arrows. Type#2 objects include extended planar objects of approximately 5 nm thickness, whose surface area was >10^4^ nm^2^. These objects were classified as layer fragments, since they occupied a confined area, and their thickness corresponded to that of the phosphatidylcholine bilayer [36]. In Figure 3, blue arrows point at the objects of this type. Layers of 5 to 7 nm thickness were classified as type#3 objects. In Figure 3, green arrows indicate objects of this type.

**Figure 4:**
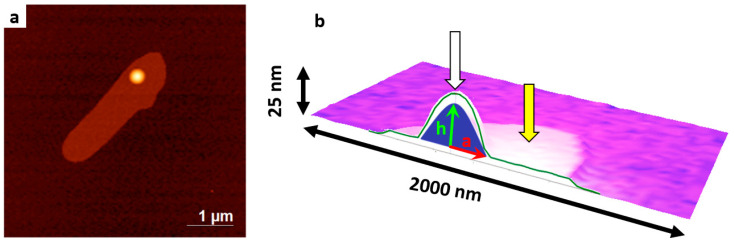
An example of type#4 object visualized on the mica surface after incubation in NFPh emulsion. (**a**) AFM image; (**b**) a schematic cross-sectional image of the object, with segment height h and radius of the segment base a specified. White arrow indicates the compact protruding part of 23 nm height; yellow arrow indicates the extended flat fragment of 2.0–2.5 nm thickness. Thin red arrow indicates the radius of the segment base *a*, and thin green arrow indicates the segment height *h*.

Type#4 objects (indicated by yellow arrows in Figure 3) were attributed to a separate category for the following reasons. First, these objects were non-stochastic, and they were observed in all the samples immediately after their preparation. Second, although these objects differed in size, their common feature was a compact protruding part of 7 to 30 nm height on an extended planar element of 2 to 5 nm thickness. Figure S2 provides an additional description of this type of objects (see Supplementary Materials File). We assume that objects of this type appeared due to fusion of several hundreds or thousands of vesicles. At that, the total number of water molecules in the inner vesicle volume and the number of phosphatidylcholine molecules remained unchanged. Figure 4 displays a zoomed-in image of this type of object and a schematic picture of each element. We calculated the number of internal water molecules within a compact segment (Figure 4b) and the total number of phosphatidylcholine molecules forming the object. The number of phosphatidylcholine molecules can be determined by measuring the total area of a planar element considering it to be a single layer with an assumption that the surface area of a single phosphatidylcholine molecule is 62 Å^2^ (see Supplementary Materials File). We have re-calculated the number of internal water molecules under the spherical segment approximation using the formula

$$V=\frac{\pi h}{6}\left({3a}^{2}+h^{2}\right),$$

where *a* is the radius of the segment base, and *h* is the segment height. 

Under the assumption that there were no losses of molecules during the fusion of monodisperse vesicles, the molar ratio of water and phosphatidylcholine molecules should be equal to that for a single vesicle. Such a ratio was calculated for the single vesicles of various diameters (see Table S1 in Supplementary Materials File). 

These calculations show that the *N*(*H_2_O*)/*N*(*PC*) ratio is ~5–7, remaining within this range for all the visualized type#4 objects. Figure S3 (see Supplementary Materials File) displays an example of the data obtained. As is demonstrated in the Supplementary Materials, this ratio is typical for vesicles of ~20 nm diameter (see Table S1 in Supplementary Materials File). Thus, type#4 objects emerge due to fusion of vesicles of ~19–21 nm size.

For type#1 objects, we also estimated vesicle size based on the AFM data. Upon the analysis of the AFM data, it was taken into consideration that NFPh deforms in the course of their interaction with the surface and can become planar [34,40,41]. This surface-bound NFPh can correspond to either single or aggregated compact objects, which were attributed to type#1 ones (Figure 3, red arrows). By determining the areas of these planar objects, one can calculate the radius of a bulky particle [31]. Assuming that the vesicle diameter in the emulsion is *D*, and the diameter of the planar structure on the surface is $D^{\prime }$, the surface area of the bilayer vesicle is

$$S=4\pi {\left(\frac{D}{2}\right)}^{2}.$$

The surface area of the planar structure is

$$S^{\prime }=\pi {\left(\frac{D^{\prime }}{2}\right)}^{2}.$$

Assuming that the bilayer vesicle and the planar single-layer structure consist of an identical number of phospholipid molecules and that $S^{\prime }=2S$, *D* can be expressed through $D^{\prime }$:

$$D=\sqrt{2}D^{\prime }/4$$

**Figure 5:**
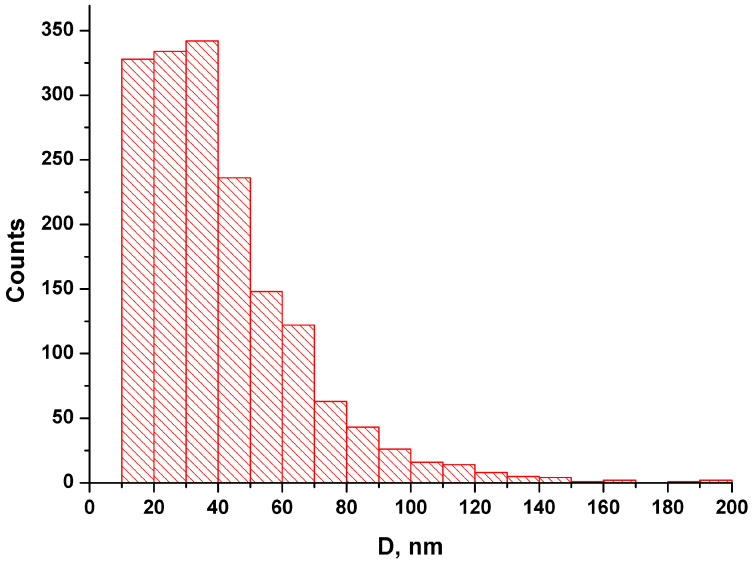
Estimation of vesicle diameter in the emulsion based on the AFM data. The histogram of distribution of reconstructed vesicle diameters *D* calculated according to the surface area of type#1 objects visualized using AFM.

In Figure 5, the results of the calculations using the formula listed above are shown in the form of a histogram of distribution of vesicle diameters *D* in the emulsion. According to the calculation, the vesicles in the emulsion have diameters within the range from 15 to 40 nm. The larger objects correspond to aggregated vesicles.

## 3. Discussion

The use of AFM for studying nanophospholipid particles implies adsorption of the target objects onto a solid substrate surface in order to allow their visualization and the determination of their morphology. Several factors can affect the adsorption of a phospholipid and, thus, the obtained results and their interpretation. One of these factors is the effect of the AFM tip, which causes the deformation of visualized vesicles during AFM scanning [42]. The other factors are the chemical nature of the surface [43], the vesicle size [35], the pH and the ionic strength of the buffer [44], and the chemical composition of the vesicles [45].

Once again, the use of AFM requires adsorption of analyzed objects on the solid substrate surface. This adsorption can be accompanied by the fusion of vesicles, distortion of their shape, etc. The fusion of phospholipid vesicles on the solid substrate surface was previously studied using AFM [35,42,46]. The fusion on the mica surface continues for a long time. According to the data obtained via AFM scanning of the substrate surface with NFPh adsorbed on it, spherical macro-objects formed on the surface on the next day after sample preparation (see Figure S4 in Supplementary Materials File), while no objects were observed on the same surface on the first day.

The AFM data obtained for the freshly prepared samples indicate that the surface contained objects formed by the fusion of various numbers of vesicles. Namely, the fusion of a large number of vesicles apparently leads to the formation of phospholipid layers and their fragments with a height equal to that of the phosphatidylcholine bilayers or multilayers. This phenomenon was reported in the literature for a number of phospholipids and has been proven using AFM and other methods [34,40,41].

The fusion of ~100–1000 vesicles gives rise to type#4 objects. Type#1 objects are formed upon the fusion of a smaller number of vesicles (they can also be represented by single vesicles). The presence of these objects likely depends on the sample preparation conditions, including humidity, incubation time of the emulsion on the substrate, etc. The sample preparation conditions used in this study have allowed us to visualize this type of objects within several hours; however, providing their stability requires additional experiments. The recorded zeta potential (ζ) value (ζ = −8.8 ± 0.8 mV) also provides the short-term stability of the phospholipid nanoparticles. According to the literature data, strongly charged particles are characterized by a high zeta-potential, which prevents fusion and provides redispersion owing to electrostatic repulsion. The absolute zeta potential value 30 mV ≤ ζ ≤ 60 mV is indicative of a high stability of nanoparticles. The values of particles’ zeta potential ζ ≥ ±30 mV indicate that a nanoparticle composition is monodisperse and aggregate-free. At ζ ≈ ±20 mV, the particles can be characterized by short-term stability. At ζ < 5 mV, they tend to aggregate rapidly. Meanwhile, it should be borne in mind that the zeta-potential is not the only indicator of stability of nanoparticles [47]. 

**Figure 6:**
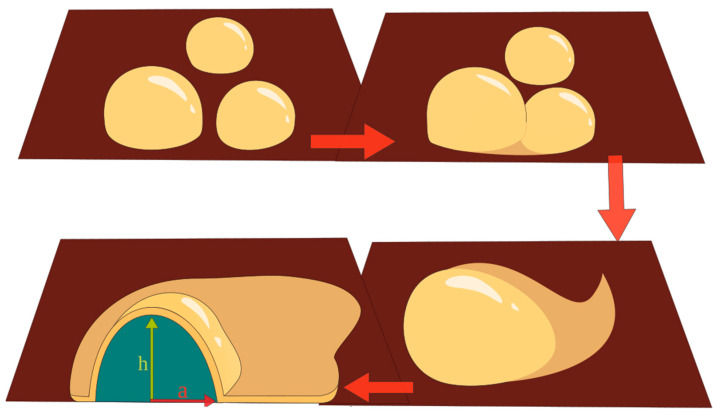
Schematic representation of the possible fusion process and type#4 object formation on mica substrate surface. Thick red arrows indicate the sequence of the fusion process stages. Thin red arrow indicates the radius of the segment base *a*, and thin green arrow indicates the segment height *h*.

We assume that vesicles in the emulsion have a spherical shape. For compact particles (type#1 and type#4), we have compared the sizes determined using DLS with those determined using AFM. According to the AFM data, this size estimation has indicated that the vesicle diameter in the emulsion is ~20 nm, which is in accordance with the DLS data. Vesicle fusion processes on the mica substrate surface are accelerated. It has been demonstrated that when a number of vesicles fuse, the internal water (or emulsion) resides in a confined area and tends toward a spherical shape (the core). Meanwhile, phosphatidyl choline molecules arrange to form a planar structure and tend to occupy the entire mica surface area. Figure 6 demonstrates a schematic representation of the possible fusion process and formation of a type#4 object on a mica substrate surface. 

The novelty of the results obtained in our study is that the number of phospholipid molecules can be determined for these objects, which mainly reside in the extended planar part of the object. Furthermore, the AFM data allow one to estimate the volume of water confined within the compact spherical part of this type of objects. Upon the use of NFPh as a drug delivery system, information about the internal volume of the vesicle can be quite useful for the estimation of the drug concentration inside the vesicle. Eventually, the AFM data for compact objects, visualized on the surface, can be used to reconstruct the diameter of particles (vesicles) in the initial volume, which is a characteristic of the NFPh.

## 4. Materials and Methods

The experiment design was as follows. An NFPh specimen was prepared using the previously developed procedure [17], and is described in Section 4.1. A distinctive feature of the NFPh studied—in comparison with the final product (the drug) [25]—was the absence of maltose. The latter acts as a cryoprotectant, which is required to perform freeze drying of the resulting composition. The NFPh was characterized using the standard methods. The size of particles in the emulsion was determined using DLS, and its physicochemical parameters (pH, light transmittance, particles’ zeta potential, and polydispersity index) were measured in parallel. Immediately after preparation, the emulsion was transferred to perform its AFM measurements. In AFM experiments, the emulsion sample was incubated on the surface of a freshly cleaved mica substrate. The surface was scanned with an atomic force microscope immediately after the preparation of the AFM specimen (mica with adsorbed emulsion particles), as well as on the next day after specimen preparation in order to estimate the stability of the visualized objects.

### 4.1. Preparation of the NFPh

Lipoid S100 (10.0 g) (Lipoid GmbH, Ludwigshafen, Germany) was suspended in 100 mL of purified water. Sodium glycyrrhizinate (1.0 g) (Vital Chem Zhuhai Co., Ltd., Zhuhai, China) was separately dissolved in 50 mL of purified water. After the dissolution, the aqueous solutions of sodium glycyrrhizinate and lipoid S100 were combined, and resuspending was carried out. The solution was brought to a final volume of 200 mL using purified water. The resulting coarse emulsion was heated to 45 °C on an Elmasonic S 300 H ultrasonic cleaner (Elma, Pforzheim, Germany), poured into the receiving container of an M110EH30K microfluidizer processor (Microfluidics, Westwood, MA, USA), and cyclic-mode homogenization was performed (seven cycles; 1000 atm; emulsion temperature, 45 °C). The resulting ultrafine emulsion was filtered using an YY30 090 00 installation (Millipore Corporation, Burlington, MA, USA) by being passed through a glass fiber prefilter (1 µm pore size) and a membrane filter (0.22 µm pore size).

### 4.2. NFPh Characterization by Macroscopic Methods

Particle size of the NFPh was determined with a Zetasizer Nano laser correlation spectrometer (Malvern Instruments Ltd., Malvern, UK) by DLS. The measured particle size range was from 0.6 to 6000 nm. Other parameters of the measurements were as follows: cell temperature, 25 °C; equilibration time, 3 min. The laser wavelength was 632.8 nm. Scattered light intensity was detected at backscattering angle of 173° and converted to the autocorrelation function (with particle size distribution of the tested sample being its result). The measurements were carried out using a 1 mL aliquot of the sample diluted tenfold with purified water and placed in a plastic cell, using the standard operating procedure (SOP).

The emulsion pH was determined using a STARTER ST300 pH meter (Corporation, Parsippany, NJ, USA). The measurements were carried out at room temperature in three technical replicates.

Light transmittance (transparency) of the NFPh emulsion was determined with an Agilent 8453 UV-visible Spectroscopy System spectrophotometer (Agilent Deutschland GmbH, Waldbronn, Germany), which was operated using HP UV Visible ChemStation v. A10.01 software (Hewlett-Packard, Wilmington, DE, USA). Distilled water was used as a reference solution, whose transmittance was measured first. Next, 3 mL of the emulsion sample was poured into the cell, and its light transmittance was measured at a wavelength of 660 nm.

The zeta potential was determined using a Zetasizer Nano ZS (Malvern Instruments Ltd., Malvern, UK) analyzer via electrophoretic light scattering. A cell containing 1 mL of test emulsion, diluted tenfold with purified water, was placed into the measuring chamber, and the specialized kit containing a dip cell (Zen 1002) was immersed into it. The measurements were carried out using the standard operating procedure (SOP). 

Statistical significance of the measured parameters in three replicas was assessed using the Student’s *t*-test. The differences were considered statistically significant at *p* ≤ 0.05.

### 4.3. Atomic Force Microscopy

A 5 µL volume of the freshly prepared NFPh emulsion, prepared as described in Section 4.1, was dispensed onto freshly cleaved mica. Mica surface (0.15 mm thick, sized 15 × 15 mm, TipsNano, Zelenograd, Russia) was used as a substrate for non-covalent adsorption.

The emulsion droplet was incubated on the mica substrate surface for 10 min. Then, the substrate was washed with 1 mL of deionized water, which was obtained using a Simplicity UV system (Millipore, Molsheim, France). The washed substrate was dried in air and subjected to AFM scanning.

The AFM images of nanosized particles were obtained with a Dimension atomic force microscope equipped with an Icon^™^ scanner (Bruker, Billerica, MA, USA). The instrument is a part of the Avogadro unique research facility (http://avo.ibmc.msk.ru/ (accessed on 1 October 2023)). Scanning was carried out in the tapping mode. A short cantilever holder was used; the measurements were conducted in air. The images were recorded using the NanoScope 9.4 software (Bruker, Billerica, MA, USA). AFM images were processed using the standard NanoScope Analysis 2.0 software (Bruker, Billerica, MA, USA), Gwyddion 2.62, and Femtoscan Online software 4.8 (LLC Scientific and Production Enterprise “Center for Advanced Technologies”, Moscow, Russia; www.nanoscopy.net/en/Femtoscan-V.shtm (accessed on 6 May 2021)).

## 5. Conclusions

The nanophospholipid composition (NFPh) has been studied using AFM. Different types of objects, formed by adsorbed NFPh vesicles, have been visualized and characterized in size. The objects visualized were divided into four different types based on their size.

One of these types of objects represented an extended phospholipid part and compact protrusion with internalized water. It has been shown that such objects have formed due to vesicle fusion. Their diameter was estimated to be 20 nm. The latter value is in good accordance with the particle size measured using DLS. 

The recovery of a single vesicle size in solution from AFM data can be used in the investigation of other phospholipid systems under the assumption that all the molecules of the initial vesicles participate in the fusion process. Thus, water and phosphatidylcholine molecules molar ratio should be equal to the molar ratio for a single vesicle.

## Data Availability

The data underlying this research can be obtained from the corresponding author upon reasonable request.

## Supplementary Materials

The following supporting information can be downloaded at: https://www.mdpi.com/article/10.3390/ijms242015338/s1. References [48,49,50,51,52,53,54,55,56] are cited in the supplementary materials.
