# Supplementary material for: Nanoform of Phospholipid Composition: Investigation of the Morphological Features by Atomic Force Microscopy
Source: Int J Mol Sci. 2023 Oct 19;24(20):15338. doi: 10.3390/ijms242015338 (PMC10607005; doi:10.3390/ijms242015338)
Supplement: Supplementary file 1 [file ijms-24-15338-s001.zip › ijms-2629057-supplementary.pdf]

Supplementary Information to:

Nanoform of Phospholipid Composition: Investigation of the Morphological Features by atomic force microscopy

by

Sergey V. Kraevsky*, Irina A. Ivanova, Sergey L. Kanashenko, Ivan D. Shumov, Ilya A. Ryazantsev, Yulia A. Tereshkina, Lyubov V. Kostryukova, Yulia A. Romashova, and Tatyana O. Pleshakova

Suppl_1. Estimation of the size of an individual vesicle of the phospholipid composition based on the data on critical micelle concentration (CMC)

The parameter of critical micelle concentration (CMC) of phospholipids (PLs) can be used to discuss the findings and roughly estimate the expected size of phospholipid nanoparticles. With regard to phospholipid-based nanosystems, CMC is a key parameter determining the state and behavior of the system. The CMC value depends on several factors. The first one is the PL fatty acid composition.

CMC is strongly dependent on both the number of fatty acids (one or two) in the PL molecule and on chain length of fatty acids [1]. Namely, the longer the hydrocarbon chains of fatty acids, the lower the CMC is. According to [1], increasing the chain length of fatty acids by a single $–CH_2-$ residue lowers the CMC value by at least half an order of magnitude.

Figure S1 illustrates the dependence between the CMC of phosphatidylcholine (PC) and the number of carbon atoms in fatty acid chains of PC known from the literature data.

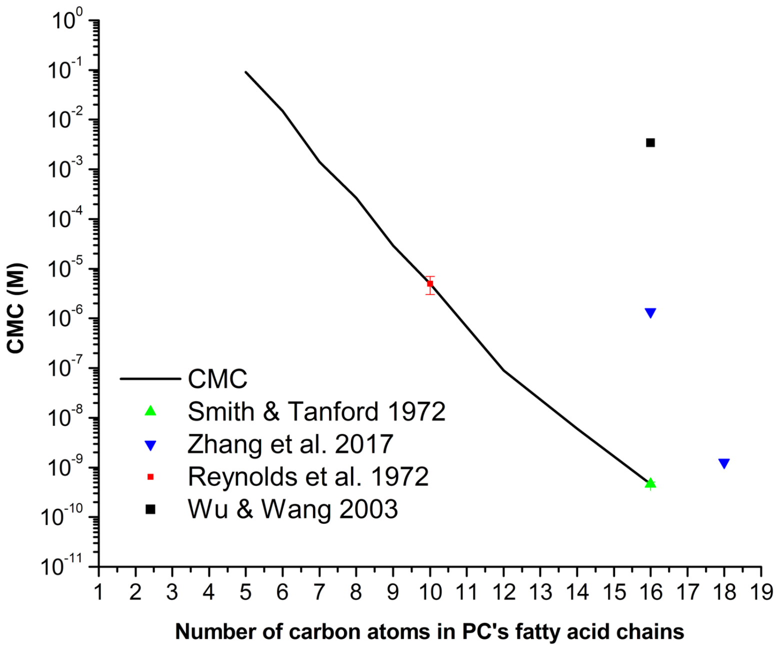

Figure S1. The CMC of PC as a function of the number of carbon atoms in its fatty acid chains. Markers show the experimental data obtained by different research groups for different types of PC. L- α -didecanoyl PC (red square) [3]; L-α-dipalmitoyl PC (green triangle) [2]; dipalmitoyl PC and distearoyl PC (blue inverted triangles) [4], and PC fraction of soy lecithin (black square) [5].

In their fundamental study, Smith and Tanford [2] reported that the CMC of synthetic L-α-dipalmitoyl-PC in water is 4.6 ± 0.5×10^{-10} M (the green marker in Figure **S1**). For a disubstituted PC with shorter carbon chains, Reynolds et al. [3] obtained the CMC value of 5.0 ± 2×10^{-6} M (the red marker in Figure **S1**). They conducted measurements in 0.02 M Tris-Cl buffer supplemented with 0.1 M NaCl, 5 mM NaN_3, and 1 mM EDTA (pH 8.3; ionic strength, 0.133). The findings obtained by Smith and Tanford, and by Reynolds et al. are consistent with the data shown in the dependence (Figure S1, black curve) [1]. Later, Zhang et al. [4] found that the CMC values of PC dissolved in phosphate buffered saline (pH 7.2) containing two palmitic or stearic acid residues were 1.36×10^{-6} M and 1.27×10^{-9} M, respectively (blue markers in Figure S1). The researchers mentioned that these values were significantly higher than the reference one obtained by Smith and Tanford and attributed this rise in the CMC to the influence of ionic strength of the buffer solution, which increases CMC. At that, a trend toward a decrease in the CMC with increasing the length of PC's fatty acid chains.

It should also be mentioned that the data obtained for PC of soybean lecithin fraction and reported by Wu and Wang [5] were several orders of magnitude higher than the values discussed above: the reported CMC value was 2.67 mg/mL (corresponding to ~ 3.4×10^{-3} M) for this fraction in water (the black marker in Figure 6). Thus, the dependence between the CMC and the length of fatty acid chains of disubstituted PC can be described as a decrease in the CMC value with increasing the length of fatty acid chains, and as an increase in CMC with increasing the ionic strength of PC solution.

In this study, the phospholipid composition is based on soybean phosphatidylcholine. Soybean PC contains a high amount of polyunsaturated fatty acids with the following fatty acid distribution: approximately 13% palmitic (C_{16}:0), 4% stearic (C_{18}:0), 10% oleic (C_{18}:1), 64% linoleic (C_{18}:2), and 6% linolenic (C_{18}:3) acid [6]. Taking into consideration that linoleic acid (18 carbon atoms per molecule) is the predominant fatty acid in this phospholipid and neglecting the effect of double bonds in a linoleic acid molecule on the CMC of phospholipid, from the dependence shown in Figure S1 one can expect that the CMC of the tested phospholipid in water will be

~ 10^{-10} M. Nevertheless, taking into account the data reported by Wu and Wang [5], there will be no surprise if in the case of a PC fraction isolated from a natural material, the CMC value will be quite high, and one should be aware that such a situation is quite possible.

In the tested phospholipid emulsion, phosphatidylcholine (PC) concentration is 47.5 mg/mL, which corresponds to 0.061 M PC (3.78×10^{19} PC molecules per mL). This concentration is one order of magnitude higher than the CMC value obtained by Wu and Wang for the phosphocholine fraction of soybean phospholipid[5]. Thus, in our case, the formation of a colloid system of phospholipid nanoparticles is expected.

Let us estimate the weight of a single phospholipid nanoparticle according to its size. The thickness of the vesicle wall formed by a phospholipid bilayer is typically ~5 nm [7–9]. Let us assume that the vesicle has a spherical shape, the outer diameter being D_{ves} = 20 nm. Then, the inner vesicle diameter will be:

$$d_{inn} = D_{ves} - 2\delta = 20 - 2 \times 5 = 10 \text{ nm}. \quad \text{(Eq. S1)}$$

The surface area of the outer and inner lipid layers of a vesicle:

$$s_{out} = \pi \langle d_{out}^2 \rangle = 1256 \text{ nm}^2$$

$$s_{inn} = \pi \langle d_{inn}^2 \rangle = 314 \text{ nm}^2. \quad \text{(Eq. S2)}$$

The surface area occupied by a single PC molecule is known to be s_0= 60 to 62 Å^2= 0.62 nm^2 [9,10]. Accordingly, the inner layer of the PC vesicle will comprise

$$N_{inn} = s_{inn}/s_0 = 523 \text{ PC molecules}, \quad \text{(Eq. S3)}$$

while the outer layer will comprise

$$N_{out} = s_{out}/s_0 = 2093 \text{ PC molecules}. \quad \text{(Eq. S4)}$$

The entire 20 nm spherical vesicle will thus comprise

$$N = N_{inn} + N_{out2} = 2616 \text{ PC molecules}. \quad \text{(Eq. S5)}$$

Given the molecular weight of PC M_w=782.1 g/mol, the weight of a single PL vesicle is

$$N \times M_w / N_A = 2616 \times 782.1 / (6.02 \times 10^{23}) = 3.4 \times 10^{-18} \text{g} = 3.4 \times 10^{-15} \text{mg} \quad \text{(Eq. S9)}$$

Thus, assuming PC concentration to be [PC] >> CMC and neglecting the number of unbound PC molecules in the emulsion, we have the number of vesicles per mL of phospholipid sample suspension: $47.5 / (3.4 \times 10^{-15}) = 1.4 \times 10^{16}$ vesicles/mL.

Table S1 lists the values characterizing the dependence between the ratio of the number of water molecules and the number of phosphatydilcholine molecules *(N(H2O)/N(PC))* on vesicle diameter D_{ves}. The value 0.62 nm^2, which characterizes the area occupied by a single PC molecule, was used to calculate the number of phosphatidylcholine molecules *N(PC)* [9,10]. The number of water molecules *N(H2O)* was calculated with allowance for the fact that one cubic nanometer contains 33 water molecules [11].

Table S1. The ratio between the number of water molecules and the number of phosphatidylcholine molecules *(N(H2O)/N(PC))* as a function of vesicle diameter D_{ves}.

Vesicle diameter D_{ves}, nm	Vesicle radius R_{ves}, nm	Vesicle surface area, nm^2	Water volume inside a vesicle V_{H2O}, nm^3	Number of phosphatidylcholine molecules *N(PC)*	Number of water molecules *N(H2O)*	*N(H2O)/N(PC)*
14	7	665	33	1073	1105	1.0
16	8	916	113	1478	3730	2.5
18	9	1218	267	1965	8842	4.5
20	10	1570	523	2532	17270	6.8
22	11	1971	904	3180	29842	9.4
24	12	2424	1436	3909	47388	12.1
26	13	2926	2143	4720	70737	15.0
28	14	3479	3052	5611	100718	17.9
30	15	4082	4186	6583	138160	21.0
32	16	4735	5572	7637	183891	24.1
34	17	5438	7234	8771	238740	27.2
36	18	6192	9198	9987	303537	30.4
38	19	6995	11488	11283	379111	33.6
40	20	7850	14130	12661	466290	36.8
42	21	8754	17148	14119	565903	40.1
44	22	9708	20569	15659	678780	43.3
46	23	10713	24416	17280	805749	46.6
48	24	11768	28716	18981	947639	49.9
50	25	12874	33493	20764	1105280	53.2

Suppl_2. Description of the AFM image of a surface region containing type#4 objects

Figure S2 displays typical AFM image of the surface predominantly containing type#4 objects that consist of a compact protruding part and an extended planar element. The AFM image of the surface was recorded immediately after sample preparation: mica contains objects adsorbed from the tested NFPh emulsion. Figure S2a displays the AFM image indicating the surface topography, while Figure S2b shows the cross-section profile shown by the line in Figure S2a. In this Figure, one can see that single compact objects (blue arrows) are visualized on the surface, being localized as extended planar fragments (green arrows). The cross-sectional line drawn through the target objects allows one not only to determine the height but also identify whether the object is isolated and whether it lies in a planar region (e.g., on the step edge). In this case, in Figure S2b, the cross-sectional profile also demonstrates that there is both an object ~ 7.5 nm high and a step where a compact object 2–3 nm high resides. The cutoff level (3 nm) is used when plotting the function of object height distribution in order to remove fragments of extended objects from the calculation and determine height for compact objects only. The results of analyzing the AFM data were used to plot the function of object height distribution $\rho(h)$ (Figure S2c)) according to which the compact portion of objects of this type mainly has size ranging from 4 to 15 nm.

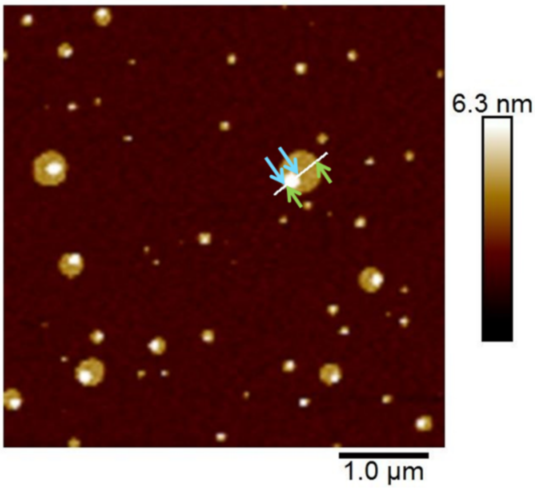

(a)

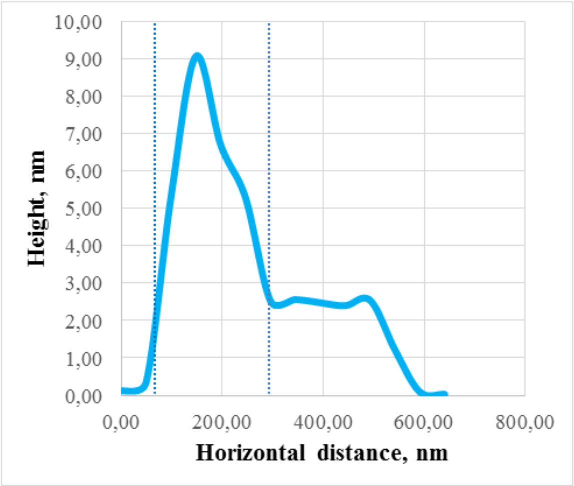

(b)

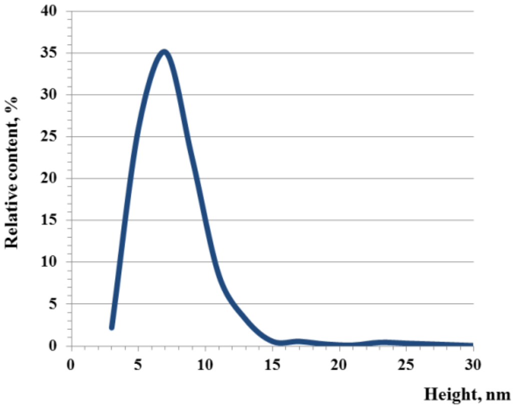

(c)

Figure S2. The results of AFM visualization of the mica surface with objects adsorbed onto it after incubation in NFPh emulsion. Demonstration of determining parameters of type#4 objects. Visualization of the surface immediately after sample preparation. An AFM image ((a) 2D; (b) the cross-sectional profile corresponding to the line in image (a); (c) a diagram of the distribution function of visualized compact objects). In image (a), blue arrows show the compact objects for which the distribution histogram (c) was plotted and green arrows show the extended parts.

Suppl_3. Calculation of the ratio between the number of phosphatidylcholine molecules and the number of water molecules inside a compact fragment for three objects classified as type#4 visualized objects

V_{int} is the volume of internal water calculated under spherical segment approximation using the formula $V = \frac{\pi h}{6}(3a^2 + h^2)$, where a is the radius of the segment base, and h is the segment height;

S_{Ph} is the area of the phospholipid layer forming an extended planar fragment of the object;

$N(H_2O)/N(PC)$ is the ratio between the numbers of water molecules in the inner vesicle volume and phosphatidylcholine molecules forming a vesicle;

D_{ves} is the diameter of an initial vesicle in the emulsion of phospholipid composition.

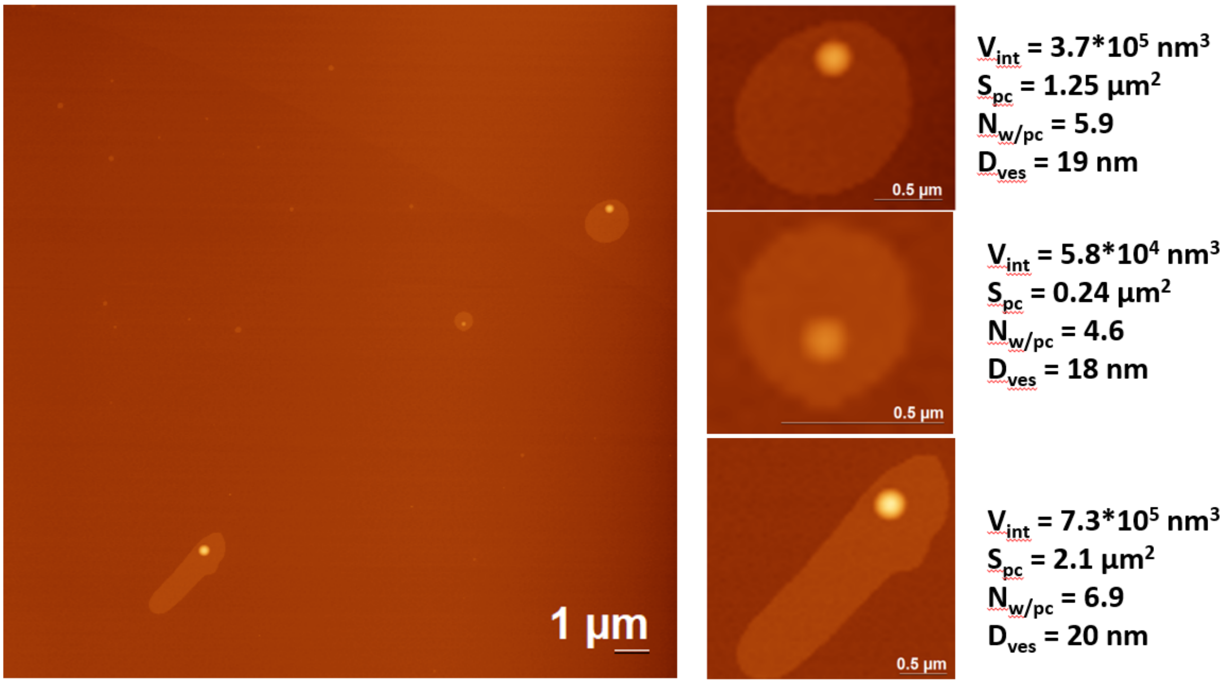

Figure S3. Left panel: an example of the AFM image of a surface region containing type#4 objects visualized after incubation in NFPh emulsion. Central panel: the zoomed-in AFM image of the objects for which an example of calculation is provided. Right panel: the results of processing the AFM data for each object: *V_{int}* is the volume of water contained in the compact protruding portion; *S_{Ph}* is the area occupied by the phospholipid layer forming an extended planar fragment of the object; *$N(H_2O)/N(PC)$* is the ratio between the number of water molecules in the inner vesicle volume and the number of phosphatidylcholine molecules forming a vesicle.

Suppl_4 Surface imaging the second day after incubating mica in NFPh emulsion

AFM imaging of the surface of samples (mica with adsorbed objects) was also carried out the next day after the samples had been prepared. Figure S4 displays the typical images of the surface recorded in this experimental series.

As demonstrated in Figure S4, a single type of objects up to 100–120 nm high are visualized on the surface. The height of the objects is one order of magnitude larger compared to that for the objects visualized on the surface during the first day of observations. Meanwhile, there are no extended objects 2–5 nm high in which compact objects had previously been observed (compare to Figure S1). The resulting data indicate that the objects adsorbed onto the surface are unstable: during the first day after sample preparation, phospholipid structures migrate along the atomically smooth mica surface and their aggregation into larger objects continues; these larger objects were visualized on the surface by AFM the next day. The AFM data are supported by the results of measuring the zeta potential, which also demonstrated that the composition is unstable under storage conditions.

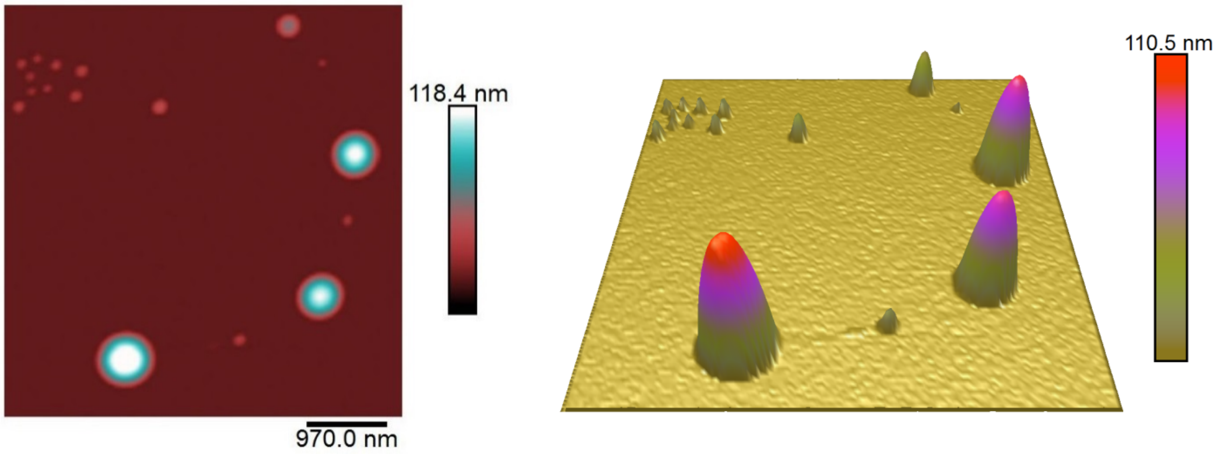

(a) **(b)**

Figure S4. The results of AFM imaging of the mica surface with adsorbed objects the next day after sample preparation. The AFM image ((**a**) 2D; (**b**) 3D) of surface topography after mica incubation in NFPh emulsion.

References.

1. Critical Micelle Concentrations (CMCs) | Avanti Polar Lipids (En-US) Available online: https://avantilipids.com/tech-support/physical-properties/cmcs (accessed on 30 August 2023).
2. Smith, R.; Tanford, C. The Critical Micelle Concentration of L-α-Dipalmitoylphosphatidylcholine in Water and Water/Methanol Solutions. *J. Mol. Biol.* **1972**, *67*, 75–83, doi:10.1016/0022-2836(72)90387-7.
3. Reynolds, J.A.; Tanford, C.; Stone, W.L. Interaction of L-Alpha-Didecanoyl Phosphatidylcholine with the AI Polypeptide of High Density Lipoprotein. *Proc. Natl. Acad. Sci. USA* **1977**, *74*, 3796–3799, doi:10.1073/pnas.74.9.3796.
4. Zhang, H.; Dudley, E.G.; Harte, F. Critical Synergistic Concentration of Lecithin Phospholipids Improves the Antimicrobial Activity of Eugenol against Escherichia Coli. *Appl. Environmental Microbiol.* **2017**, *83*, e01583, doi:10.1128/AEM.01583-17.
5. Wu, Y.; Wang, T. Soybean Lecithin Fractionation and Functionality. *J. Amer. Oil Chem. Soc.* **2003**, *80*, 319–326, doi:10.1007/s11746-003-0697-x.
6. Thomas, A.H.; Catalá, Á.; Vignoni, M. Soybean Phosphatidylcholine Liposomes as Model Membranes to Study Lipid Peroxidation Photoinduced by Pterin. *Biochim. Biophys. Acta (BBA) - Biomembranes* **2016**, *1858*, 139–145, doi:10.1016/j.bbamem.2015.11.002.
7. Nir, S.; Bentz, J.; Wilschut, J.; Duzgunes, N. Aggregation and Fusion of Phospholipid Vesicles. *Progress in Surface Sci.* **1983**, *13*, 1–124, doi:10.1016/0079-6816(83)90010-2.
8. Kucerka, N.; Pencer, J.; Sachs, J.N.; Nagle, J.F.; Katsaras, J. Curvature Effect on the Structure of Phospholipid Bilayers. *Langmuir* **2007**, *23*, 1292–1299, doi:10.1021/la062455t.
9. Parente, R.A.; Nir, S.; Szoka, F.C. PH-Dependent Fusion of Phosphatidylcholine Small Vesicles. Induction by a Synthetic Amphipathic Peptide. *J. Biol. Chem.* **1988**, *263*, 4724–4730, doi:10.1016/S0021-9258(18)68843-X.
10. Reviakine, I.; Brisson, A. Formation of Supported Phospholipid Bilayers from Unilamellar Vesicles Investigated by Atomic Force Microscopy. *Langmuir* **2000**, *16*, 1806–1815, doi:10.1021/la9903043.
11. Nibali, V.C.; Maiti, S.; Saija, F.; Heyden, M.; Cassone, G. Electric-field induced entropic effects in liquid water. *J. Chem. Phys.* **2023**, *158 (18)*, 184501. 2023). https://doi.org/10.1063/5.0139460.
